# The Frequency of Adrenal Insufficiency in Adolescents and Young Adults with Thalassemia Major versus Thalassemia Intermedia in Iran

**DOI:** 10.4084/MJHID.2015.005

**Published:** 2015-01-01

**Authors:** Sara Matin, Masoud Ghanei Jahromi, Zohreh Karemizadeh, Sezaneh Haghpanah, Vincenzo De Sanctis, Ashraf Soliman, Javad Dehbozorgian, Zahra Majd, Narges Rezaei, Mehran Karimi

**Affiliations:** 1 Department of Pediatrics, Shiraz University of Medical Sciences, Shiraz, Iran; 2 Department of Anesthesiology, Jahrom University of Medical Science, Jahrom, Iran; 3 Metabolic and Endocrinology Research Center, Shiraz University of Medical Sciences, Shiraz, Iran; 4 Hematology Research Center, Shiraz University of Medical Science, Shiraz, Iran.; 5 Pediatric and Adolescent Outpatient Clinic, Quisisana Private Accredited Hospital, Ferrara (Italy); 6 Department of Pediatrics, Alexandria University Children’s Hospital, Alexandria, Egypt.

## Abstract

**Background:**

Endocrine dysfunction is not uncommon complication in patients with transfusion-dependent thalassemia and is thought to occur as a consequence of excessive iron overload. The primary objective of this study is to determine the frequency of adrenal insufficiency in patients with thalassemia major and thalassemia intermediate.

**Methods:**

This cross-sectional study was done at the Shiraz University of Medical Sciences, Shiraz, Southern Iran, in 2013. One hundred and ninety patients were divided into two groups; thalassemia major(TM) and thalassemia intermediate (TI) groups. We measured 8 AM serum cortisol, ACTH and ferritin concentrations in all patients.

**Results:**

The mean age of the TM and TI group were 22.5±5.7 and 23.8±6 years, respectively. 90 patients (47.4%) were splenectomized, 34 (36.2%) with TM and 56 (58.2%) with TI (p :<0.001). The median and interquartile range of serum ferritin levels were 2184±3700 ng/ml and 437±443ng/ml in TM and TI respectively (p< 0.001). Three patients with TM (1.6%) had low basal cortisol and ACTH levels. However, their cortisol response to ACTH stimulation was normal.

**Conclusions:**

Low basal concentrations of cortisol and ACTH occurred in 1.6% of our adolescents young adult patients with TM suggesting a central defect in cortisol secretion at the basal state. However, cortisol response to standard – dose ACTH was normal in all patients with TM and TI.

## Introduction

β-thalassemias are a group of hereditary blood disorders characterized by anomalies in the synthesis of the beta chains of hemoglobin resulting in variable phenotypes, ranging from severe anemia to clinically asymptomatic individuals. Three main forms have been described: thalassemia major(TM) thalassemia intermedia (TI) and thalassemia minor. Individuals with TM usually present within the first two years of life with severe anemia, requiring regular red blood cell (RBC) transfusions.^1^

The life expectancy of children and adolescents with TM and TI has markedly increased, due to improvement of the quality and techniques of blood transfusion and new development of effective oral iron chelators.^1^ However, TI patients still develop iron overload, despite the lack of need for blood transfusions, because of increased intestinal iron absorption.^2^–^5^ Moreover, various endocrine abnormalities have been described in patients with TM and TI and most reports incriminate iron overload as an important factor in the development of target-organ dysfunction.^6^,^7^

Iron overload is toxic to parenchymal cells because it generates free radicals and induces oxidative stress causing damage to biomolecules, including lipids, proteins, and DNA.^1^,^3^

Therefore, regular surveillance of these patients is essential for early detection and management of possible complications, such as: heart failure and arrhythmias, chronic liver diseases, endocrine problems (hypogonadism, hypothyroidism, diabetes mellitus, hypoparathyroidism and adrenal insufficiency), growth failure, osteoporosis and thrombophilia.^1^,^6^ In TM, the prevalence of adrenal insufficiency (AI) is variable because of the variable degree and duration of iron overload and not standardized cutoff cortisol values for diagnosing cortisol deficiency. In addition, the prevalence of AI has not been investigated well in adolescent patients with TI. Although, most studies have revealed intact pituitary adrenal axis in TM, several recent studies reported a significant prevalence of subclinical “biochemical” AI, ranging from 18–45% in these patients.^8^–^15^ AI is either primary, due to deposition of excess iron in the adrenal gland or secondary due to the toxic effects of iron in the pituitary gland.^16^–^18^ The combined measurement of early morning serum cortisol and plasma ACTH separates patients with primary adrenal insufficiency from healthy individuals and from those with secondary disease.^19^ While the diagnosis of overt adrenal failure is generally straightforward, the identification of asymptomatic patients with subtle dysfunction of the hypothalamic-pituitary-adrenal (HPA) axis is still a diagnostic challenge.

We assessed basal and stimulated cortisol secretion in relation to the degree of iron overload in 190 consecutive adolescent patients with TM and TI.

## Subjects and Methods

This study included all patients who were referred to the thalassemia clinic, Hospital and Outpatient Clinic, affiliated with the Shiraz University of Medical Sciences. The patients divided into two groups according to their diagnosis: TM and TI. All patients with TM were on regular blood transfusion every 2–4 weeks. TI patients were transfusion independent.

The diagnosis of thalassemia was based on complete blood count (CBC), Hb electrophoresis and clinical history.

Inclusion criteria were all TM and TI patients aged 10 years and above.

Exclusion criteria included: history of any infection or stress such as surgery in the past four weeks, congestive heart failure, thyroid dysfunction, hepatic impairment, uncontrolled diabetes mellitus, and/or taking any medications that may have an effect on adrenal function. Four-hundred-ninety TM and 196 TI patients over 10 years old were eligible. From these patients, 190 consecutive patients were enrolled in our study.

A venous blood sample was collected from all patients at 8 AM for measuring serum ferritin, cortisol, and ACTH concentrations.

### Assessment of adrenal function

A 2.5 mL sample of fasting venous blood withdrawn from the participants was added to CBC tube contained EDTA anticoagulant. Plasma, centrifuged immediately, was preserved in −20 °C in the freezer, then carried by cold box to the laboratory to test ACTH level. Furthermore, 3 mL clot blood was also withdrawn from each patient. The serum was immediately separated without hemolysis, preserved in −20 °C in the freezer and then, carried by cold box to the laboratory to test cortisol level.

The ACTH and cortisol levels were measured by use of an electrochemiluminescence immunoassay (ECLIA) method (Radim Diagnostics, Pomezia- Rome, Italy). Normal range of cortisol at 7–10 A.M. was 6.2–19.4 μg/dL and 2.3–11.9 μg/dL at afternoon, while that for corticotropin (ACTH) was 7.2–63.3 pg/mL.

### Differentiation between primary and secondary adrenal insufficiencies

Analysis of the results was considered as follows:

1. A low plasma cortisol levels (< 6.2 μg/dL – 10th percentile), measured between 8.00–9.00 am, in the face of high ACTH levels (*i.e.* > 100 pg/mL) suggested primary AI.^19^,^20^
2. Inappropriately normal or low ACTH levels (< 7.2 pg/mL), in the presence of low cortisol level (< 6.2 μg/dL – 10th percentile), suggested secondary AI.^21^
3. Failure to increase cortisol levels above 18 μg/dL at 30–60 minutes post corticotropin intravenous (i.v.) stimulation test indicated adrenal insufficiency.^22^

### Standard dose ACTH stimulation test

The ACTH stimulating test was performed with 250 *μ*g synthetic ACTH 1–24, cosyntropin, tetracosactin, Synacthen, as i.v. bolus followed by measurement of serum cortisol 30 and 60 min after the injection (CINACT ampoule).

Fasting serum ferritin was measured by Electro Fluorescent Assay (ELFA) method, Mini Vidas machine (bio Merieux SA, France).

### Statistical analyses

Statistical analyses were performed with SPSS Software (SPSS: An IBM Company, version17.0, IBM Corporation, Armonk, NY, USA). Test of normality was performed by Shapiro-Wilk Test for serum ferritin, ACTH, and cortisol levels. Mann-Whitney test was used for comparison of serum ferritin, ACTH, and cortisol levels between TM and TI patients. Student t-test was used for comparison of age between the two groups. Comparison of qualitative data was done by Chi-square test. A p value <0 .05 was considered as statistically significant.

### Ethical aspects

The study was performed in accordance with provisions of the Declaration of Helsinki and Good Clinical Practice guidelines and was approved by Medical Ethics Committee of the Shiraz University of Medical Sciences. Written informed consent was obtained from the patients or their parents.

## Results

Ninety-four TM and ninety-six TI patients were investigated. The mean age of the TM and TI group were 22.5±5.7 and 23.8±6 years, respectively. No significant age difference was found between the two groups (p:0.15).

In the TM group, 55 subjects (58.5%) were females and 39 males (41.5%). In the TI group, 41 (42.7%) were women. All patients with TM have been on iron chelators with deferoxamine (DFO) (70%), combined DFO and deferiprone (DFP) (10%) or deferasirox (DFX) (20%).

The compliance to DFO and DFX iron chelators were fair and good, respectively. Among the study subjects, 90 (47.4%) had splenectomy, 34 (36.2%) with TM and 56 (58.2%) with TI, respectively (p:0.001). The median and interquartile range of serum ferritin levels in TM and TI groups were 2184±3700 ng/ml and 437±443 ng/ml respectively (p< 0.001). (Table 1)

In general, an early morning (8 am) plasma cortisol level lower than 6.2 μg/dL – (10th percentile) is suggestive for primary AI, whereas a value higher than 15 μg/dL makes the diagnosis highly unlikely. Therefore, we performed an ACTH stimulation test only in 3 patients (2 males and a female; 1.6%) with low basal levels of cortisol (Table 2).

Patient 1 was on treatment with DFO and had dilated cardiomyopathy with normal left ventricular ejection fraction (LVEF). Patient 2 has short stature and pubertal delay with mild systolic and diastolic cardiac dysfunction. He has been on iron chelation therapy with DFO plus DFP and L-thyroxine therapy for primary hypothyroidism. Patient 3, while on treatment with DFX, presented with diabetes mellitus, dilated cardiomyopathy and normal LVEF. None of the 3 TM patients were on sex hormone replacement therapy or had received corticosteroids treatment.

Patients 2 and 3 had poor compliance with iron chelation therapy.

The plasma cortisol response at 60 minutes post ACTH injection should reach ≥ 18 *μ*g/dl in normal people.^22^ In our TM patients, the cortisol responses to ACTH stimulation test resulted in normal range (Table 2).

All patients with TI had normal basal serum ACTH and cortisol concentrations.

The median of ACTH concentrations did not differ significantly between patients with TM and TI (p: 0.072, Table 1). The median serum ferritin and basal serum cortisol levels were significantly higher in TM versus TI patients (p<0.001, Table 1 ).

## Discussion

A large body of evidence has emerged linking severe iron overload with increased vulnerability to endocrine dysfunction in patients with thalassemia.^1^,^2^ Thalassemia patients, requiring continuous blood transfusion, suffer from iron overload, with a resultant increase in free non–transferrin-bound iron (NTBI), and iron accumulation in vital organs. In fact, the NTBI is rapidly taken up by liver and other tissues. A particular portion of NTBI is the chelatable labile plasma iron (LPI), which is not found in healthy individuals. The LPI is the most toxic component; its toxicity is due to an high reduction-oxidation (redox) potential, that generates oxygen-free radicals such as superoxide anions, which damages DNA, proteins, and membrane lipids in the cells.^23^ Another source of iron accumulation results from increased duodenal iron absorption due to decreased expression of hepcidin, the central regulator of iron homeostasis.^24^,^25^ Iron has a marked affinity for the different endocrine glands.^26^–^29^ The pituitary, thyroid and parathyroid gland, as well as the endocrine pancreas, are variably affected in these patients.^30^–^32^ It appears that the hypothalamic pituitary adrenal axis is the least affected among the others in thalassemic patients. However, because of the histological and imaging evidence of iron deposits in the adrenal cortex^33^,^34^ the potential risk of AI in these patients carries a significant risk that requires early diagnosis.

Adrenal insufficiency can be primary or secondary. Primary AI occurs when the adrenal glands are damaged and cannot produce enough of the adrenal hormone cortisol. The adrenal hormone aldosterone may also be lacking.^17^ Secondary adrenal insufficiency occurs when the pituitary gland fails to produce enough adrenocorticotropin (ACTH). If ACTH output is too low, cortisol production drops. Eventually, the adrenal glands can shrink due to lack of ACTH stimulation.

Our study showed that all adolescents and young adults with TI had normal ACTH- the cortisol axis. Only in three patients with TM the cortisol and ACTH concentrations were low. However, their cortisol response to 250 *μ*g synthetic ACTH was normal.

Different cut-offs for normal serum cortisol secretion have been proposed, and the most reliable criterium appear to be a cortisol peak greater than 18–20 *μ*g/dl after ACTH stimulation to exclude AI.^35^ However, this test cannot be able to detect recent onset or mild forms of secondary AI. However, a normal cortisol response does not exclude secondary AI, because the adrenal glands, which have not yet undergone significant atrophy, can still respond to high dose ACTH stimulation.^36^

It is well known that the sensitivity of ACTH stimulation test to pick up mild adrenal insufficiency improves when using the low-dose of cosyntropin (1 μg ACTH given intravenously); but, this may result in a higher false-positive rate. In addition, the lack of a commercially available 1-μg dose may represent another potential error.

Although 250 mcg ACTH test (owing to a massive dose of ACTH) is not sensitive for diagnosis of partial secondary adrenal insufficiency, the basal cortisol value during the standard-dose test has in most clinical situations a diagnostic accuracy close to that of a low-dose of ACTH test.^37^,^38^

Our 3 TM patients with low basal secretion of cortisol and ACTH (secondary AI) were asymptomatic. It is well known that the generalized weakness; tiredness and fatigability are, in the early phase of the adrenocortical insufficiency, transient and appearing only after increased physical or psychical stress. They become gradually more intensive and in more advanced stages of chronic adrenocortical insufficiency.^21^,^36^

A standard-dose of 250 μg cosyntropin is useful for excluding primary AI in those with low cortisol level during screening. A cortisol response higher than 18 μg/dL at 30 minutes after a standard-dose ACTH confirms adequate cortisol secretion. Most individuals with normal adrenal function achieve much higher cortisol levels at 60 minutes after cosyntropin injection.

It should be also noted that a large variety of total cortisol assays is commercially available with considerable differences in specificity, sensitivity, accuracy, precision and reproducibility of many of these assays. Some of these assays appear to overestimate or underestimate actual cortisol levels and, as such, hamper the correct diagnosis of relative AI.^39^

These findings raise several interesting, relevant issues. Hematologists need to be more vigilant about endocrine complications in thalassemia. Whenever possible, cases of AI should be interpreted and managed in consultation with a pediatric or adult endocrinologist, because adrenal insufficiency usually is not in thalassemia an acute event but results from gradual decline in pituitary function, due to iron overload.

Deficiency of cortisol level (secondary AI) with intact renin-angiotensin-aldosterone system can cause adrenal crisis if it is severe or patients are in acute illness.

Therefore, we recommend a systemic testing of the adrenal function prior infection, trauma, surgical intervention or other stress and at regular yearly interval in TM patients with iron overload especially in those with other endocrinopathies.

For patients with proven AI, family education and stress steroids during times of illness, injury or surgery are imperative help reduce the morbidity and mortality associated with this serious complication. Recovery from other endocrinopathies may be possible in thalassemia by using intensive iron chelation therapy; however, this issue has not been studied in cases of AI.

## Conclusions

Our findings support the small prevalence of AI in adolescents and young adults with TM. All patients with TI have normal basal cortisol and ACTH secretion.

An early morning (8 am) plasma cortisol level lower the 10° percentile is suggestive for adrenal insufficiency, whereas a value higher than 15 μg/dL makes the diagnosis highly unlikely. Cortisol levels of intermediate range may be seen in patients with primary, secondary or tertiary adrenal insufficiency. In those cases and in patients with low basal cortisol and standard response to ACTH a strict collaboration with the endocrinologist is needed.

## Figures and Tables

**Table 1:** Comparison of median and interquartile range of the serum ferritin (ng/ml), cortisol (μg/dL) and ACTH (pg/ml) between TM and TI groups.

Variable	Group	N	Median	Interquartile range	P-Value
Ferritin level	TM	94	2184	3700	<0.001
	TI	96	437	443	
Cortisol level	TM	94	17.34	6.51	<0.001
	TI	96	13.07	5.69	
ACTH level (pg/ml)	TM	94	16.57	27	0.072
	TI	96	13.54	23	

**Table 2:** Age, sex, serum ferritin (ng/ml), basal ACTH (pg/ml), cortisol (ng/ml ) basal and after ACTH stimulating test (30 and 60 min).

Patient	Age	Sex	Serum ferritin	Basal cortisol and ACTH	Cortisol after ACTH (30 min)	Cortisol after ACTH (60 min)
1	23	female	968	0.702 < 1	22.2	28.54
2	11	male	>6400	6.02 3.89	27.48	32.78
3	25	male	>6400	3.57 4.08	26.3	30.2

Note: normally the plasma cortisol response at 30–60 minutes post ACTH injection should reach ≥ 18 μg/dl.

## References

[1] Galanello R, Origa R (2010). Beta-thalassemia. Orphanet J Rare Dis.

[2] Musallam KM, Cappellini MD, Taher AT (2013). Iron overload in ß-thalassemia intermedia: an emerging concern. Curr Opin Hematol.

[3] Musallam KM, Taher AT, Rachmilewitz EA (2012). ß-thalassemia intermedia: a clinical perspective. Cold Spring Harb Perspect Med.

[4] Tanno T, Miller JL (2010). Iron loading and overloading due to ineffective erythropoiesis. Adv Hematol.

[5] Hentze MW, Muckenthaler MU, Andrews NC (2004). Balancing acts: molecular control of mammalian iron metabolism. Cell.

[6] (1995). Multicentre study on prevalence of endocrine complications in thalassaemia major. Italian working group on endocrine complications in non-endocrine diseases. Clin Endocrinol (Oxf).

[7] Belhoul KM, Bakir ML, Saned MS, Kadhim AM, Musallam KM, Taher AT (2012). Serum ferritin levels and endocrinopathy in medically treated patients with ß thalassemia major. Ann Hematol.

[8] Sklar CA, Lew LQ, Yoon DJ, David R (1987). Adrenal function in thalassemia major following long-term treatment with multiple transfusions and chelation therapy. Evidence for dissociation of cortisol and adrenal androgen secretion. Am J Dis Child.

[9] Jaruratanasirikul S, Tanchotikul S, Wongcharnchailert M, Laosombat V, Sangsupavanich P, Leetanaporn K (2007). A low dose adrenocorticotropin test (1 microg ACTH) for the evaluation of adrenal function in children with B-thalassemia receiving hypertransfusion with suboptimal iron-chelating therapy. J Pediatr Endocrinol Metab.

[10] Srivatsa A, Marwaha RK, Muraldharan R, Trehan A (2005). Assessment of adrenal endocrine function in Asian thalassemics. Indian Pediatr.

[11] Mehrvar A, Azarkeivan A, Faranoush M, Mehrvar N, Saberinedjad J, Ghorbani R, Vossough P (2008). Endocrinopathies in patients with transfusion-dependent beta-thalassemia. Pediatr Hematol Oncol.

[12] Vogiatzi MG, Macklin EA, Trachtenberg FL, Fung EB, Cheung AM, Vichinsky E, Olivieri N, Kirby M, Kwiatkowski JL, Cunningham M, Holm IA, Fleisher M, Grady RW, Peterson CM, Giardina PJ, Thalassemia Clinical Research Network (2009). Differences in the prevalence of growth, endocrine and vitamin D abnormalities among the various thalassaemia syndromes in North America. Br J Haematol.

[13] Scacchi M, Danesi L, Cattaneo A, Valassi E, Pecori Giraldi F, Radaelli P, Ambrogio A, D’Angelo E, Mirra N, Zanaboni L, Cappellini MD, Cavagnini F (2010). The pituitary-adrenal axis in adult thalassaemic patients. Eur J Endocrinol.

[14] Elsedfy HH, El Kholy M, Tarif R, Hamed A, Elalfy M (2011). Adrenal function in thalassemia major adolescents. Pediatr Endocrinol Rev.

[15] Soliman AT, Yassin M, Majuid NM, Sabt A, Abdulrahman MO, De Sanctis V (2013). Cortisol response to low dose versus standard dose (back-to-back) adrenocorticotrophic stimulation tests in children and young adults with thalassemia major. Indian J Endocrinol Metab.

[16] Costin G, Kogut MD, Hyman CB, Ortega JA (1979). Endocrine abnormalities in thalassemia major. Am J Dis Child.

[17] Pasqualetti P, Colantonio D, Collacciani A, Casale R, Natali G (1990). Circadian pattern of circulating plasma ACTH, cortisol, and aldosterone in patients with beta-thalassemia. Acta Endocrinol (Copenh).

[18] Pasqualetti P, Collacciani A, Colantonio D, Casale R, Natali G (1990). Circadian rhythm of pituitary-adrenal axis in thalassemia. Recenti Prog Med.

[19] Wilkinson CW, Pagulayan KF, Petrie EC, Mayer CL, Colasurdo EA, Shofer JB, Hart KL, Hoff D, Tarabochia MA, Peskind ER (2012). High prevalence of chronic pituitary and target-organ hormone abnormalities after blast-related mild traumatic brain injury. Front Neurol.

[20] Oelkers W, Diederich S, Bahr V (1992). Diagnosis and therapy surveillance in Addison’s disease: rapid adrenocorticotropin (ACTH) test and measurement of plasma ACTH, renin activity, and aldosterone. J Clin Endocrinol Metab.

[21] Siafarikas A (2010). Addison disease. Diagnosis and initial management. Aust Fam Physician.

[22] Jacobson L (2005). Hypothalamic-pituitary-adrenocortical axis regulation. Endocrinol Metab Clin North Am.

[23] Dixon SJ, Stockwell BR (2014). The role of iron and reactive oxygen species in cell death. Nat Chem Biol.

[24] Schmidt PJ, Fleming MD (2014). Modulation of hepcidin as therapy for primary and secondary iron overload disorders: preclinical models and approaches. Hematol Oncol Clin North Am.

[25] Chauhan R, Sharma S, Chandra J (2014). What regulates hepcidin in poly-transfused ß-Thalassemia Major: Erythroid drive or store drive?. Indian J Pathol Microbiol.

[26] Kishimoto M, Endo H, Hagiwara S, Miwa A, Noda M (2010). Immunohistochemical findings in the pancreatic islets of a patient with transfusional iron overload and diabetes: case report. J Med Invest.

[27] Matsushima S, Tsuchiya N, Fujisawa-Imura K, Fujisawa-Imura K, Hoshimoto M, Takasu N, Torii M, Ozaki K, Narana I, Kotani T (2005). Ultrastructural and morphometrical evaluation of the parathyroid gland in iron-lactate-overloaded rats. Toxicol Pathol.

[28] Lu JP, Hayashi K (1994). Selective iron deposition in pancreatic islet B cells of transfusional iron-overloaded autopsy cases. Pathol Int.

[29] Iancu TC, Ward RJ, Peters TJ (1990). Ultrastructural changes in the pancreas of carbonyl iron-fed rats. J Pediatr Gastroenterol Nutr.

[30] Argyropoulou MI, Kiortsis DN, Metafratzi Z, Bitsis S, Tsatoulis A, Efremidis SC (2001). Pituitary gland height evaluated by MR in patients with beta-thalassemia major: a marker of pituitary gland function. Neuroradiology.

[31] Wood JC, Noetzl L, Hyderi A, Joukar M, Coates T, Mittelman S (2010). Predicting pituitary iron and endocrine dysfunction. Ann N Y Acad Sci.

[32] Noetzli LJ, Panigrahy A, Mittelman SD, Hyderi A, Dongelyan A, Coates TD, Wood JC (2012). Pituitary iron and volume predict hypogonadism in transfusional iron overload. Am J Hematol.

[33] Bhamarapravati N, Na-Nakorn S, Wasi P, Tuchinda S (1967). Pathology of abnormal hemoglobin diseases seen in Thailand. I. Pathology of beta-thalassemia hemoglobin E disease. Am J Clin Pathol.

[34] Drakonaki E, Papakonstantinou O, Maris T, Vasiliadou A, Papadakis A, Gourtsoyiannis N (2005). Adrenal glands in beta-thalassemia major: magnetic resonance (MR) imaging features and correlation with iron stores. Eur Radiol.

[35] Hockings GI, Nye EJ, Grice JE, Jackson RV (1997). Short synacthen test versus insulin stress test for the assessment of hypothalamo-pituitary axis: controversy revisited again. Clin Endocrinol (Oxf).

[36] Dorin RI, Qualls CR, Crapo LM (2003). Diagnosis of adrenal insufficiency. Ann Intern Med.

[37] Mayenknecht J, Diederich S, Bahr V, Plöckinger U, Oelkers W (1998). Comparison of low and high dose corticotropin stimulation tests in patients with pituitary disease. J Clin Endocrinol Metab.

[38] Annane D, Sebille V, Charpentier C, Bollaert PE, François B, Korach JM, Capellier G, Cohen Y, Azoulay E, Troché G, Chaumet-Riffaud P, Bellissant E (2002). Effect of treatment with low doses of hydrocortisone and fludrocortisone on mortality in patients with septic shock. JAMA.

[39] Cohen J, Ward G, Prins J, Jones M, Venkatesh B (2006). Variability of cortisol assays can confound the diagnosis of adrenal insufficiency in the critically ill population. Intensive Care Med.

